# Analysis of Carbon-Based Microelectrodes for Neurochemical Sensing

**DOI:** 10.3390/ma12193186

**Published:** 2019-09-28

**Authors:** Felicia S. Manciu, Yoonbae Oh, Abhijeet Barath, Aaron E. Rusheen, Abbas Z. Kouzani, Deidra Hodges, Jose Guerrero, Jonathan Tomshine, Kendall H. Lee, Kevin E. Bennet

**Affiliations:** 1 Department of Physics, University of Texas at El Paso, El Paso, TX 79968, USA; jaguerrero9@miners.utep.edu; 2 Border Biomedical Research Center, University of Texas at El Paso, El Paso, TX 79968, USA; 3 Department of Neurologic Surgery, Mayo Clinic, Rochester, MN 55905, USA; Oh.Yoonbae@mayo.edu (Y.O.); Barath.Abhijeet@mayo.edu (A.B.); Rusheen.Aaron@mayo.edu (A.E.R.); Lee.Kendall@mayo.edu (K.H.L.); 4 School of Engineering, Deakin University, Waurn Ponds, VIC 3216, Australia; abbas.kouzani@deakin.edu.au; 5 Department of Electrical and Computer Engineering, University of Texas at El Paso, El Paso, TX 79968, USA; drhodges@utep.edu; 6 Division of Engineering, Mayo Clinic, Rochester, MN 55905, USA; Tomshine.Jonathan@mayo.edu

**Keywords:** scanning electron microscopy (SEM), confocal Raman spectroscopy, fast-scan cyclic voltammetry (FSCV), carbon fiber, boron-doped diamond thin film

## Abstract

The comprehensive microscopic, spectroscopic, and *in vitro* voltammetric analysis presented in this work, which builds on the well-studied properties of carbon-based materials, facilitates potential ways for improvement of carbon fiber microelectrodes (CFMs) for neuroscience applications. Investigations by both, scanning electron microscopy (SEM) and confocal Raman spectroscopy, confirm a higher degree of structural ordering for the fibers exposed to carbonization temperatures. An evident correlation is also identified between the extent of structural defects observed from SEM and Raman results with the CFM electrochemical performance for dopamine detection. To improve CFM physico-chemical surface stability and increase its mechanical resistance to the induced compressive stress during anticipated *in vivo* tissue penetration, successful coating of the carbon fiber with boron-doped diamond (BDD) is also performed and microspectroscopically analyzed here. The absence of spectral shifts of the diamond Raman vibrational signature verifies that the growth of an unstrained BDD thin film was achieved. Although more work needs to be done to identify optimal parameter values for improved BDD deposition, this study serves as a demonstration of foundational technology for the development of more sensitive electrochemical sensors, that may have been impractical previously for clinical applications, due to limitations in either safety or performance.

## 1. Introduction

The characteristics of carbon fiber microelectrodes (CFMs) based biosensors for measuring extracellular concentrations of neurochemicals and other biological analytes, in real-time and with high accuracy, have been of continuous scientific interest for the past few decades [1,2,3,4,5,6,7,8,9]. A variety of electrochemical recording techniques have employed such sensors, including constant-potential amperometry and fast-scan cyclic voltammetry (FSCV) [10,11,12,13,14,15]. Since sensitivity in monitoring changes in neurochemical concentrations, *in vitro* and *in vivo*, strongly depends on the microelectrode material, on its design, and on its fabrication processes, a large body of research has been directed towards such considerations [1,2,3,4,5,6,7,8,9,10,11,12,13,14,15]. Carbon-based materials, such as pyrolytic graphite, carbon fibers (CFs), glassy carbon (GC), pitch-based graphitic foams, nanographite ribbons, fullerenes, carbon nanotubes (CNT), and doped diamond are still considered to be the most suitable candidates because of their biocompatible, conductive, and mechanical properties [1,2,3,4,5,6,7,8,9,10,11,12,13,14,15]. Thus, from the time when CF was invented by Joseph Swan in 1860 [16], it has been continually researched and its properties improved for multiple applications. While, originally, natural biomaterials such as cotton and bamboo were used as precursors, current research efforts and manufacturing development of CFs have enabled their commercial mass production from polyacrylonitrile (PAN)-based white fiber. The high tensile strength-to-weight ratio, excellent thermal and chemical resistance, and high thermal and electrical conductivity of PAN-based CFs make them superior to other available candidates [17,18,19].

While generally effective for *in vivo* electrochemical sensing, the surfaces of CFs have been further functionalized and modified to increase their selectivity and sensitivity in detecting a specific analyte in the presence of other biointerferents, or in monitoring multiple compounds [20,21,22,23,24,25,26]. The carbon surface, which is a disordered graphite layer with a low specific activity, can be easily modified and its properties tuned to achieve the desired outcomes. Thus, coating the fiber surface with polymers, enzymes, redox hydrogels, nanotubes, or metallic nanoparticles are examples of methods that have been commonly employed [20,21,22,23,24,25,26]. Among the most investigated selectivity problems is the interference between dopamine and ascorbic acid. Negatively charged polymer coatings, such as overoxidized polypyrrole (oPPy) and Nafion have been employed to repel and eliminate, at physiological pH, the anionic ascorbate and to improve the detection of cationic dopamine [25,26]. Other methods that have been applied consist of etching the CF surface electrochemically or by flame treatment [27,28,29,30]. In the design of CFMs, the commonly used process entails encapsulating CFs with typical diameters of 7 to 10 μm into glass or silica capillaries that are further attached to metallic wires at one end, and sealed in place with silver conductive paste. The other exposed end of the carbon fiber is sealed with 1:1 ethylendiamine epoxy resin, left to dry for a couple of hours, and then trimmed to the desired length [12,13,14,15,31].

Originally, however, commercially available CFs were never designed for electrochemical and clinical purposes, but rather for use in various manufacturing industries. For industrial applications, hundreds or thousands of micron-sized fibers, covered with an adhesion material, were wound into bundles (tows) to produce a thread used in weaving carbon fiber fabrics. In such applications, the important physical characteristic is the tensile strength of the fiber. The micro-sized fibers were never optimized for compressive stress (as opposed to tensile stress), an important characteristic for safe electrode penetration of tissue. In addition, uniform electronic properties or chemical composition were never of critical importance in such manufacturing, but are necessarily important factors for clinical applications. These manufacturing design issues present a number of practical problems in the laboratory. First and foremost, commercially sourced fibers, while immensely strong when bundled together, are very delicate when used individually under the compressive bending load that is encountered in penetrating tissue. In addition, since commercial fibers were never intended for use in electrochemical sensing, different commercial sources of these fibers can vary widely in composition, electrical stability, and chemical surface adsorption properties, characteristics critical in their use as electrochemical sensors. Sourced CFs also exhibit variable capacitive background currents, and relatively rapid and variable drifts in the baseline current detected by FSCV. Overall, the present lack of uniformity and lack of CFs produced specifically for electrochemical detection of neurochemicals *in vivo* has led to qualitatively and quantitatively different responses to the same analytes, confounding analysis and repeatability among research studies.

However, boron-doped diamond (BDD) thin film coatings on metallic rods or on silicon substrates have been used as alternatives for improving stability, limiting background noise, and reducing microelectrode fouling [9,15,32,33]. While improved physico-chemical stability has been reported for these BDD-based microelectrodes, a lower sensitivity to neurotransmitter detection was also found, through FSCV analysis, in comparison to CFMs. To meet the requirements of efficient electrochemical detection, the quality of BDD microelectrodes depends not only on the boron doping level, but also on the choice of the substrate used, as the lattice mismatch between the substrate and the BDD thin film can induce an unwanted strained structure on the latter [32,33]. Thus, deposition of BDD on carbon fibers has been proposed to reduce CF surface degradation [34,35]. This approach can also reduce the number of defects in the BDD thin film, as an unstrained structure is anticipated; no lattice mismatch occurs, because both the substrate and the thin film are carbon-based materials. On the other hand, difficulty in such deposition has also been reported [34,35], as the atomic hydrogen needed for diamond growth will etch the carbon substrate.

With the objective of achieving more accurate detection and monitoring of neurotransmitters at concentrations specific to physiological levels, we present in this work a comprehensive microscopic, spectroscopic, and electrochemical analysis of CFMs fabricated from PAN-based CFs. To improve the CFM surface stability and potentially increase its performance under compressive stress, BDD deposition on CFs is also microspectroscopically investigated here.

## 2. Materials and Methods

### 2.1. Carbon Fibers

The CFs analyzed in this work consist of three commercially available PAN-based fibers produced by CARBON NEXUS in Geelong, Australia. CARBON NEXUS, one of the largest CF manufacturing companies, is a worldwide distributor. A low heating temperature of 225–285 °C, that affects the thermal stabilization process, was applied to the CF labeled as sample 1. Samples 2 and 3 were processed at low (450–950 °C) and high (1000–1500 °C) carbonization temperatures, respectively.

### 2.2. Scanning Electron Microscopy System

The scanning electron microscopy (SEM) measurements were performed with a Helios G4 UXe DualBeam focused ion beam FIB/SEM system (Thermo Fisher Scientific, Hillsboro, OR, USA). Sections from the three different CF samples were placed over a carbon tape for fiber surface investigations or attached to a Si substrate for cross-section analysis. The micromanipulator accessory of the imaging system was employed for moving the samples and mounting them vertically to the Si substrate. A Xe^+^ source was used to cross-section mill the samples. An Elstar + UC and an Xe^+^ electron column were employed to acquire the SEM images with accelerating voltage and emission set to 1 keV and 13 pA or to 2 keV and 25 pA, rendering the desired magnification and resolution. A 750 V stage bias was applied for high-resolution images.

### 2.3. Confocal Raman System

The Raman measurements were acquired at ambient conditions in a backscattering geometry with an *alpha 300RAS WITec* system (WITec GmbH, Ulm, Germany) equipped with a 1024 × 127 pixel, Peltier cooled CCD camera. The 532-nm excitation of a frequency-doubled neodymium-doped yttrium–aluminum–garnet (Nd: YAG) laser that was restricted to a power output of a few mW, and a 100× objective lens with a numerical aperture of 0.9, were used. To obtain Raman mapping images, selected areas of the samples were raster scanned under the laser beam and arrays of the Raman spectra were recorded with an acquisition time of 50 milliseconds for each pixel and an overall time per image of a few minutes. The *WITec Control* software was utilized for controlling the piezo stage during the scans, as well as for data acquisition. Appropriate background subtractions were performed for all Raman spectra.

### 2.4. Fast Scan Cyclic Voltammetry System

*In vitro* FSCV measurements were conducted using the WINCS (Wireless Instantaneous Neurotransmitter Concentration Sensing) Harmoni system manufactured at the Mayo Clinic in Rochester, Minnesota. This system combines four independent FSCV channels with four independent electrical stimulation channels, as detailed elsewhere [36]. A standard triangular waveform at 10 Hz, with the holding potential of −0.4 V, peak potential of 1.3 V, repetition time of 100 ms, and scan rate of 400 V/s, was used to exploit adsorption and overoxidation of dopamine on the carbon fiber, for optimal sensitivity [37,38,39]. Dopamine (DA), the neurotransmitter used for these measurements, was dissolved in distilled water at a stock concentration of 1 mM, diluted with TRIS buffer (15 mM tris aminomethane, 3.25 mM KCl, 140 mM NaCl, 1.2 mM CaCl_2_, 1.25 mM NaH_2_PO_4_, 1.2 mM MgCl_2_, and 2.0 mM Na_2_SO_4_, with the pH adjusted to 7.4), and preserved with 0.1 M perchloric acid. From this stock solution, dilutions were made in TRIS buffer for the desired final concentrations, before starting the flow-injection experiments. All pharmacological agents were purchased from Sigma Aldrich (St. Louis, MO, USA). A flow-injection analysis system, which consists of a syringe pump (Harvard Apparatus, Holliston, MA, USA) that directs a buffer solution through a Teflon tube to a 6-port injection valve (Rheodyne, Rohnert Park, CA, USA) was employed for these measurements. An injection valve, which was controlled by a 12 V DC solenoid, was also used to transport the analyte from an injection loop to an electrochemical flow-cell, at a rate of 2 mL/min.

### 2.5. Boron-Doped Diamond Deposition System

The boron-doped diamond thin film was grown by hot-filament assisted chemical vapor deposition (CVD) using a custom-built reactor at the Mayo Clinic, in Rochester, Minnesota. A mixture of methane (2 sccm), hydrogen (178 sccm), and trimethylborane (TMB) (20 sccm; 1000 ppm TMB in hydrogen, Voltaix Products, Branchburg, NJ, USA) was introduced into the chamber, at a total chamber pressure of 20 Torr. The temperatures of 2000 °C for the filament and 800 °C for the substrate were monitored with a Spectrodyne DFP 2000 optical pyrometer and an Omega Engineering type K thermocouple, respectively, and kept constant during the 30 min film deposition. A Proportional Integral Derivative (PID) software-based control loop was employed for this procedure. Prior to CF (of about 7 μm in diameter) insertion into a quartz capillary, the CF was abraded by immersion in a slurry mixture of 100 nm diamond powder (Engis, 105 West Hintz Road, Wheeling, IL, USA) and isopropyl alcohol.

## 3. Results and Discussion

### 3.1. Microscopic and Spectroscopic Characterizations of Carbon Fibers

**Figure 1:**
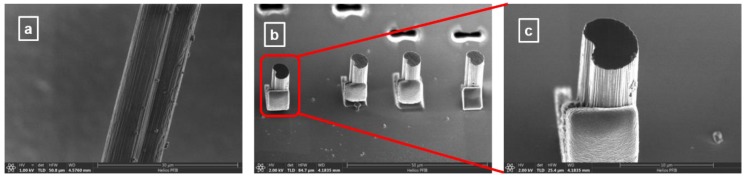
(**a**–**c**) Side view and cross-sectional SEM images of carbon fibers. For cross-sectional images mounting on Si substrates and fiber trimming were performed.

To provide a comprehensive assessment of material morphological progression during the CF manufacturing process at different temperatures, we first investigated the samples by SEM. Since no obvious differences were observed for CF surfaces, only a representative SEM surface image is presented in Figure 1a for a sample carbonized at high temperature. Small surface imperfections and irregularities can be seen in this figure of a pristine CF, as well as many longitudinal grooves, inherent in PAN-precursors. Thus, the fiber exhibits, overall, a relatively smooth surface, except for these randomly distributed granular imperfections. The characteristic quasi-oval, bean-like shape of PAN-based fibers is also observable in Figure 1b,c, where cross-sectional SEM images are shown, at different resolutions. As mentioned above, due to the flexibility, and therefore, the ease of bending these fibers, mounting on Si substrates and appropriate trimming to about 100 μm lengths were performed for such investigations.

**Figure 2:**
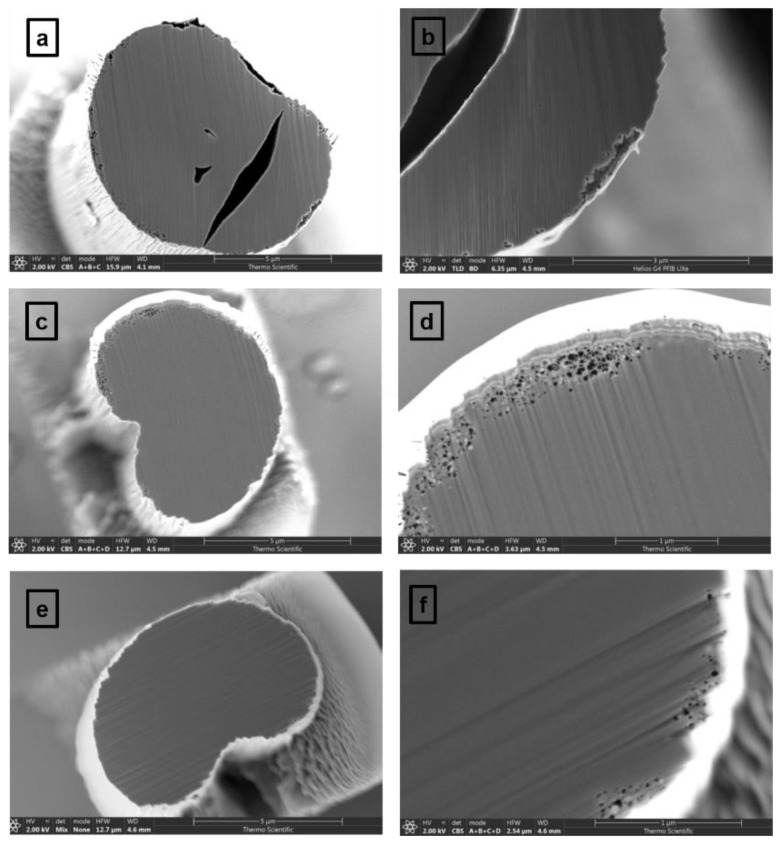
Cross-sectional SEM images, at different resolutions, of carbon fibers: (**a**,**b**) sample 1 exposed to a thermal stabilization process (225–285 °C); (**c**,**d**) sample 2 carbonized to low temperatures (450–950 °C); and (**e**,**f**) sample 3 carbonized to high temperatures (1000–1500 °C).

The quality of the fibers is further investigated through the higher resolution cross-sectional SEM images that are presented in Figure 2a–f. There are visible differences between the fiber morphologies regarding the presence and magnitude of defects; especially noticeable is the CF exposed only to a thermal stabilization process (i.e., sample 1). The SEM images associated with this sample, and presented in Figure 2a,b at different magnifications, reveal quite large hollow spaces throughout the fiber, with sizes ranging from 1–2 microns to almost the whole fiber diameter. A CF of much more improved quality is observed in Figure 2c,d, associated with sample 2, with randomly distributed defects only near the fiber surface. Their sizes are also much smaller, averaging only about 10–20 nm. Thus, a dramatic decrease in the content of defects occurs when the fiber starts the carbonization process; fibers treated to temperatures less than 500 °C can be expected to be of low quality. The cross-sectional SEM images corresponding to sample 3, which are presented in Figure 2e,f, demonstrate a further decrease in the number and average size of defects for CF carbonized at high temperature. However, only a slight improvement in fiber quality is achieved when carbonization treatment at a high versus a low temperature is applied. These observations are in agreement with previously reported results on the effects of thermal treatment on the microstructure of CFs [40], which reveal that during heat treatment, the cyclization, dehydrogenation, and oxidation reactions of the PAN polymer chains occur at a faster rate in the core of the fiber than at its surface. They also emphasize the critical importance of the thermal stabilization process and its influence on the fiber heterogeneity along the radial direction.

**Figure 3:**
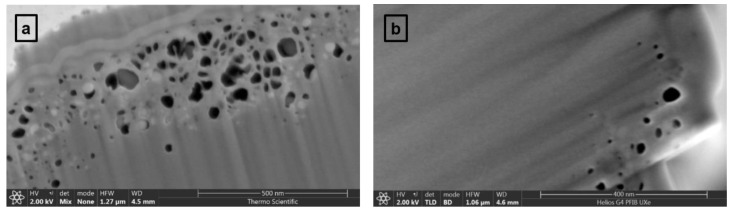
(**a**,**b**) High resolution SEM images of samples 2 and 3 enabling visualization of nanoscale defects near CF surfaces.

In addition, evidence of different cross-linking mechanisms in the core and skin regions of the fiber is provided in Figure 3a,b, where very high-resolution SEM images of samples 2 and 3 are presented. Not only do these images enable a direct comparison between the sizes and density of defects at fiber surfaces, but they also allow a visualization of nanoscale features. A higher surface porosity is observed for sample 2 than for sample 3, the latter corresponding to increased carbonization temperature.

**Figure 4:**
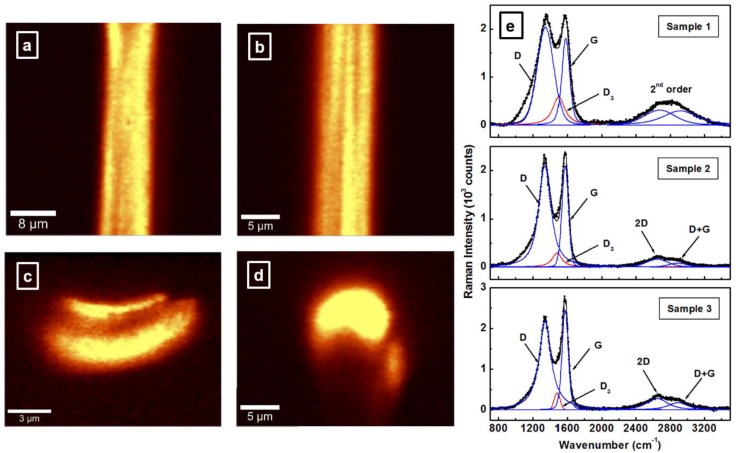
(**a**,**b**) Surface confocal Raman mapping images of samples 1 and 3, respectively. (**c**,**d**) Cross-sectional confocal Raman mapping images of samples 1 and 3, respectively. (**e**) Integrated Raman spectra of the three samples, as labeled. For clarity of vibrational assignments, the spectra were deconvoluted with Lorentz fitting lines.

Complementary analysis of CFs by confocal Raman microscopy is also presented in Figure 4a–e. A well-established characterization method in studying carbon-based materials, Raman technique has traditionally played an important role in providing information on sp^2^ or sp^3^ hybridization in materials, existence of chemical impurities, defects, and other crystal disorders, such as induced strain [4,6,7,8,9,15,19,32,33,34,35,41,42,43]. As compared with the previous images of conventional SEM, the surface and cross-sectional confocal Raman mapping images presented in Figure 4a–d show regions that do not lie within the focal plane of the optics and that are therefore not well defined. Nevertheless, longitudinal striations along the CF surface are observable in Figure 4b, as well as the large internal defect in the cross-sectional image of sample 1 (see Figure 4c). Since nanoscale voids are beyond the current optical resolution capability, they cannot be visualized in the cross-sectional images of samples 2 and 3 (for the current laser excitation of 532 nm, the resolution limit is about 350 nm). Therefore, only a representative confocal image is presented in Figure 4d for these samples, which reveals, again, the bean-like shape characteristic of PAN-based CFs. This shape is hardly distinguishable in Figure 4c associated with sample 1, due to the substantial internal defects of this sample.

Definite structural differences between the samples can be observed in Figure 4e, where the integrated Raman spectra (the averages of tens of thousands of accumulated Raman mapping spectra) associated with the three samples are shown. For additional accuracy, deconvolutions into Lorentz band shapes of the important features, such as the first and the second order vibrational modes, were also performed. Besides the G band assigned to the E_2g_ vibrational mode and present in all graphitic structures [41,42,43], which is centered at 1575 cm^−1^, the peak of the recognized defect-induced electron-hole “inter-valley” double resonance process that activates transverse optical phonon modes close to the zone boundary K point, the D band, is also observed in these spectra, at 1344 cm^−1^. An obvious decrease in the full width at half maximum (FWHM) is seen for the D band when the CFs are exposed to carbonization versus stabilization temperatures, from 227 cm^−1^ for sample 1, to 173 cm^−1^ and 171 cm^−1^ for samples 2 and 3, respectively. In addition to a similar decrease of its FWHM from 107 cm^−1^ (for sample 1) to 87 ± 2 cm^−1^ (for samples 2 and 3), the G band also shows a trend of intensity increase. This trend suggests that by employing the intensity ratio of the D and G bands, I_D_/I_G_, which is commonly used as an indicator for estimating the degree of structural disorder in carbon-based materials and of average crystal planar domain size, further discrimination between the merits of samples 2 and 3 can be achieved. The decrease in this ratio from 1.13 for sample 1, to 1.0 for sample 2, to a final value of 0.89 for sample 3, indeed, confirms the superiority of sample 3. However, the need for fitting a new Raman feature, D_3_, around 1490 ± 5 cm^−1^ (red colored line) to correctly fit the spectral shape between the D and the G bands implies the persistence of some disordered structure. This D_3_ band, which does not have any apparent maximum, has been attributed to the presence of amorphous carbon in the analysis of CFs [42,43]. Since existence of unwanted amorphous carbon impurities at CF surfaces has already been confirmed in our previous SEM measurements (see Figure 1a), the necessity of having the D_3_ feature also demonstrates good agreement between the Raman and SEM assessments. On the other hand, the decreases in the intensity and broadness of this band in the Raman spectra indicate a less defective material at higher temperature treatments, again corroborating our SEM results.

Examination of the second order Raman bands, such as the 2D (G’) and D + G bands, can provide additional information about the CF quality. For example, although not very well defined, these bands are observed around 2650 cm^−1^ and 2890 cm^−1^, respectively, for samples 2 and 3. Only a very broad band is detected for sample 1, suggesting, once more, an induced structural disorder that is quite large in this case. However, for consistency, we still fit this broad feature in the Raman spectrum of sample 1 with the two lines corresponding to 2D and D + G bands. As an indicator of a lesser degree of three-dimensional ordering in this sample, no specific band labeling is provided, as it is in the Raman spectra of samples 2 and 3.

### 3.2. Fast Scan Cyclic Voltammetry Analysis

**Figure 5:**
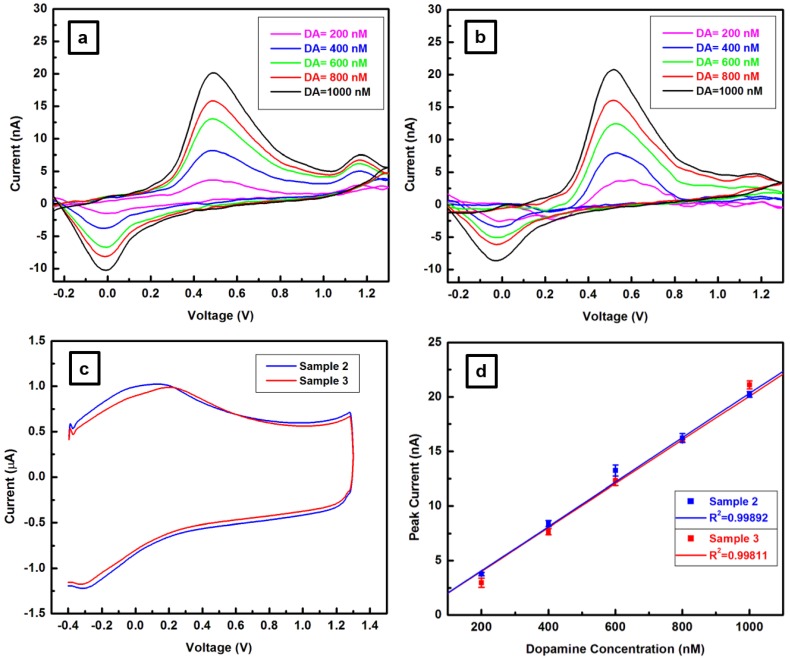
(**a**,**b**) Cyclic voltammograms at 200, 400, 600, 800 and 1000 nM dopamine (DA) for carbon fiber microelectrodes (CFMs) fabricated from samples 2 and 3 CFs, respectively, as labeled. (**c**) Shape and amplitude of the background current for the CFMs associated with (**a**,**b**). (**d**) Average detected oxidation peak current showing a linear increase for both CFMs, with a correlation coefficient of about 1.0 based upon three trials at each DA concentration.

The performance of the CFs in *in vitro* neurotransmitter detection is further investigated by FSCV, and the results are presented in Figure 5a–d. For these measurements, CFMs were made from the three sets of CFs following a design described elsewhere [12,14,31]. Prior to CFM exposure to testing, the tip of each CF was trimmed with a scalpel to a length of approximately 100 μm, in order to maintain a similar active surface area for all CFMs. A flow-cell injection system, as described above, was used for the calibration of each electrode to known concentrations of DA. For this process, the CFM was placed in a flowing stream of TRIS buffer, and the DA was injected as a bolus at concentrations of 200, 400, 600, 800 and 1000 nM. A triangle-wave potential from −0.4 to 1.3 V at 10 Hz was applied for each scan, with a scan rate of 400 V/s. Because the current recorded during each potential sweep yields a combination of capacitive and Faradaic (oxidation/reduction) currents, a sufficient number of potential sweeps were averaged before stimulation to yield a baseline template that was subtracted from every subsequent sweep to obtain Faradaic currents corresponding to the unique electrochemical signal of the neurotransmitter investigated. Thus, the heights of the peaks in the background subtracted voltammograms presented in Figure 5a,b for samples 2 and 3, respectively, are directly proportional to the concentrations of DA oxidized and reduced at the CFM surface. These voltammetric curves for DA concentrations of 200, 400, 600, 800, and 1000 nM exhibit the usual set of reduction and oxidation current peaks around 0.6 V and −0.1 V, respectively [12]. No significant difference in sensitivity to DA detection (n = 3 each concentration, paired *t*-test, p = 0.32, t = 1.132, df = 4) is evident between the two electrodes. Similar electrochemical characteristics in terms of background current are also observed in the corresponding results shown in Figure 5c. The performance of the microelectrodes, which is presented in Figure 5d, demonstrates a linear increase in the detected oxidation peak current with increasing DA concentration. A correlation coefficient of about 1.0 (R^2^ = 0.99892 for sample 2 and R^2^ = 0.99811 for sample 3) is obtained for both CFMs. All the CFMs made of sample 1 showed neither background current nor DA response, implying that this sample has no electrical conductivity. While this outcome is not surprising, based on the extent of defects observed for this sample in our previous SEM and Raman results, it also substantiates the importance and need for a detailed material analysis prior to CFM fabrication.

### 3.3. Confocal Raman Microscopy Investigation of BDD Coated Carbon Fiber

**Figure 6:**
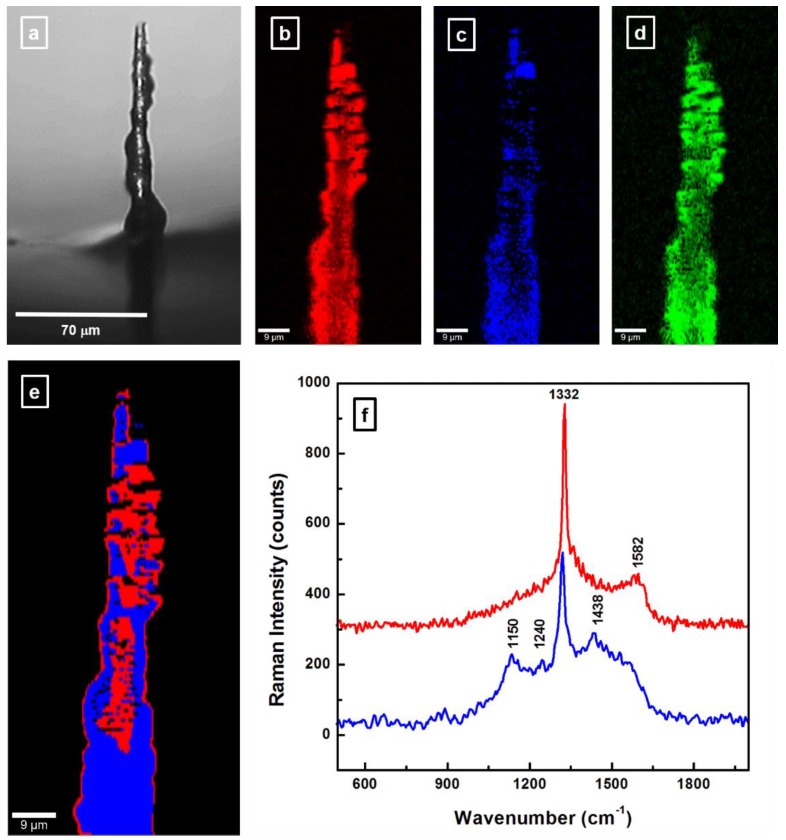
(**a**) Optical image of a boron-doped diamond (BDD) coated CF. (**b**–**d**) Confocal Raman mapping images of diamond (red), boron incorporation (blue), and sp^2^ carbon content (green), respectively. (**e**) Confocal Raman mapping image of the BDD coated CF performed with *Cluster Analysis* software. (**f**) Raman spectra associated with each cluster in image (**e**) and only in vibrational regions of interest. The same color code is maintained for the image and for the spectra.

Reproducibility of the results over time is essential for long-term usage of CFMs. However, since CF surface degradation by electrochemical etching during FSCV measurements has already been reported [15], in an attempt to increase the CF surface stability, fiber coating with BDD was also performed in this study, and the microspectroscopic results are presented in Figure 6a–f. A carbon fiber carbonized at a higher temperature (i.e., sample 3) was chosen as substrate for this process. A non-uniform deposition is seen in the optical image presented in Figure 6a, with aggregates of different sizes randomly distributed over the fiber surface. An estimation of the diameter of the felt CF at its narrower width demonstrates the absence of any significant occurrence of surface sp^2^ bond etching by the atomic hydrogen gas precursor that is essential for diamond growth; a similar CF diameter of about 7 µm can be observed in this image. Thus, the addition of boron assists with the growth process of the thin film coating, as previously reported [34,35].

The investigation of the film constituents by confocal Raman microscopy, which is presented in Figure 6b–d, confirms the achievement of BDD deposition. Selective Raman features, such as the vibrational line of diamond at 1332 cm^−1^, the broad, weak band around 1220 cm^−1^ attributed to boron incorporation into the diamond lattice, and the graphitic G band around 1580 ± 5 cm^−1^ were considered for performing these confocal Raman mapping images. For visualization, these three constituents were labeled using the following pseudo-colors: red for diamond (Figure 6b), blue for BDD (Figure 6c), and green for graphitic carbon (Figure 6d). A dominant color overlap between red and green, as well as preferential incorporation of boron (blue color) is observed in these images. This overlapping is expected due to the close spectral proximity of the Raman signatures for the sp^3^ and sp^2^ carbon hybridizations (i.e., diamond-associated sharp Raman feature at 1332 cm^−1^ overlaps with the broad D band at 1350 cm^−1^). Consequently, we present in Figure 6e a different approach for assessing potential CF morphological changes, still through confocal Raman mapping. The acquisition of this image was performed using the *Cluster Analysis* package of the *WITec Project Plus* software, which enables sorting of the 22,500 hyperspectral data set into subsets, according to spectral similarity. The reason behind maintaining the same red and blue pseudo-colors for these clusters and for the constituents was to highlight again the incidence of diamond formation and that of boron incorporation into the carbon material. The Raman vibrational lines associated with the sp^2^ carbon substrate is already incorporated in these subsets of spectra. To allow association of spectroscopic data with the specific microscale regions in Figure 6e, we present in Figure 6f the graphs corresponding to each cluster, while maintaining the same color code for the image and for spectra. While a successful coating with diamond is confirmed by the presence of the 1332 cm^−1^ Raman peak in both spectra of Figure 6f, this is not the case for boron incorporation, which, besides being preferential, also induces distortion within the graphite layer planes. This remark is supported by an obvious decrease in the G band intensity at 1585 cm^−1^ (in the red line spectrum), which becomes just a shoulder of the broad D_3_ band at 1438 cm^−1^ (in the blue line spectrum); the latter band corresponding to amorphous carbon, as discussed above. Furthermore, a comparison between the Raman spectra of pristine CF (see Figure 4e) and felt CF (red line spectrum in Figure 6f) reveals that there is a noticeable reduction in the amount of sp^2^ carbon due to coating with diamond alone. This statement is based again on the intensity decrease of the G band, which is further affected by boron addition. Finally, the Raman features at 1150 and 1240 cm^−1^, which are associated with boron incorporation into sp^2^ and sp^3^ carbon, respectively, demonstrate preferential boron assimilation throughout the entire CF. The very small intensity of the latter vibration substantiates that only a small proportion of boron is incorporated into the diamond lattice.

## 4. Conclusions

Potential for improvements in CFMs, based on a comprehensive understanding of the structure/property relationships of the employed material still remains. This is true even for structural CFs manufactured for industrial applications, which are far more developed compared with the conductive CFs needed for *in vivo* electrochemical applications. In vivo implantable CMFs require flexibility and strength under induced compressive strain from tissue penetration, consistent conductivity over the length of the fiber, and durability against surface degradation. These CF properties have not been widely studied by carbon fiber manufacturers, given their priority of structural performance at low cost. Since development in these areas would be of significant importance to biological analysis, a comprehensive microscopic, spectroscopic, and voltammetric analysis of CFs is presented in this work. The current results demonstrate an evident correlation between the structural defects observed from SEM and confocal Raman investigations and the performance of the CFMs in FSCV measurements. To improve the physico-chemical stability of the CFM surface, coating of the CF with BDD is also successfully performed and analyzed here. The absence of shifts in the position of the Raman line characteristic of diamond validates the growth of an unstrained thin-film coating. Since development of flexible, more sensitive and robust CFM biosensors is envisioned, this study contributes to specifically tailoring CFs for *in vivo* neurochemical recording in clinical applications. However, due to the complexity and unique challenges of this task, more work needs to be done to identify optimal parameters for improved uniformity of BDD deposition. Not only is the quality of the applied thin-film coating important for enhancing CFM conductivity, but also for increasing the biosensor mechanical resistance to *in vivo* embedding.
